# The network structure of paranoia in the general population

**DOI:** 10.1007/s00127-018-1487-0

**Published:** 2018-02-09

**Authors:** Vaughan Bell, Ciarán O’Driscoll

**Affiliations:** 1 Division of Psychiatry, University College London, 6th Floor, Maple House, 149 Tottenham Court Road, London, W1T 7NF UK; 2 South London and Maudsley NHS Foundation Trust, London, UK; 3 North East London NHS Foundation Trust, London, UK

**Keywords:** Psychosis, Schizophrenia, Delusion, Network, Epidemiology

## Abstract

**Purpose:**

Bebbington and colleagues’ influential study on ‘the structure of paranoia in the general population’ used data from the British National Psychiatric Morbidity Survey and latent variable analysis methods. Network analysis is a relatively new approach in psychopathology research that considers mental disorders to be emergent phenomena from causal interactions among symptoms. This study re-analysed the British National Psychiatric Morbidity Survey data using network analysis to examine the network structure of paranoia in the general population.

**Methods:**

We used a Graphical Least Absolute Shrinkage and Selection Operator (glasso) method that estimated an optimal network structure based on the Extended Bayesian Information Criterion. Network sub-communities were identified by spinglass and EGA algorithms and centrality metrics were calculated per item and per sub-community.

**Results:**

We replicated Bebbington’s four component structure of paranoia, identifying ‘interpersonal sensitivities’, ‘mistrust’, ‘ideas of reference’ and ‘ideas of persecution’ as sub-communities in the network. In line with previous experimental findings, worry was the most central item in the network. However, ‘mistrust’ and ‘ideas of reference’ were the most central sub-communities.

**Conclusions:**

Rather than a strict hierarchy, we argue that the structure of paranoia is best thought of as a heterarchy, where the activation of high-centrality nodes and communities is most likely to lead to steady state paranoia. We also highlight the novel methodological approach used by this study: namely, using network analysis to re-examine a population structure of psychopathology previously identified by latent variable approaches.

**Electronic supplementary material:**

The online version of this article (10.1007/s00127-018-1487-0) contains supplementary material, which is available to authorized users.

## Introduction

On a population level, paranoia is present as a spectrum that ranges from low-level paranoid ideation to frank paranoid delusions and includes two key components: unfounded ideas of harm and the idea that the harm is intended by others [1]. Using data from an online survey, Freeman et al. [2] first proposed that paranoia has a structure, noting that the endorsement of rarer, more severe items on a measure of paranoid thoughts predicted the greater number of items endorsed overall, suggesting that more severe paranoia rested on a ‘base’ of less severe paranoid thinking.

Bebbington et al. [3] reported a more rigorous test of this idea using data gathered with clinical assessments on a representative sample of the UK population, which was collected as part of the British National Psychiatric Morbidity Survey conducted in the year 2000 [4]. They found evidence for a similar hierarchy of paranoia that stretched into the clinical range, and additionally used confirmatory factor analysis, latent class analysis and factor mixture modelling, to look for latent variables that might represent underlying components of paranoia that formed part of the ‘paranoia hierarchy’—identifying interpersonal sensitivities, mistrust, ideas of reference and ideas of persecution as key components. Methodologically, the analysis techniques used by the researchers involved varying degrees of testing against pre-specified models—from testing against specific factor structures specified *apriori—*to being relatively model free although with a significant number of parameters specified in advance.

In recent years, a new approach to understanding psychopathology called network analysis has emerged [5–7] which is agnostic as to whether latent variables underlie syndromes as common causes and also allows for causal interactions between symptoms to explain symptom groupings. For example, it is possible that the symptoms of depression are directly caused by common underlying aetiological factors but it is also possible that some symptoms arise as a *consequence* of other symptoms: poor concentration in depression could be a consequence of poor sleep.

On a fundamental level, network analysis examines partial correlations between symptom measures controlled for every other variable in the network, providing plausible candidates for causal interactions. Each symptom is represented as a node and each partial correlation as an edge, allowing the mathematics of graph theory to be used to further analyse the structure and reveal candidates for the most influential symptoms and clusters of symptoms in networks [5, 6]. Measures of ‘centrality’ provide plausible hypotheses for the most influential nodes that mediate and sustain relationships in the wider network and potentially indicate points of intervention for ‘dismantling’ networks of self-sustaining symptoms [7, 8]. It is also possible to derive network communities (equivalent to network sub-clusters) that reveal sub-networks that make up the larger network structure (e.g. [9]) indicating a mid-level structure between individual nodes and the complete network.

Network analysis also has the advantage of being model free at the level of psychopathology and generated solely from the data. In addition, it has the practical benefit of involving fewer researcher degrees of freedom in the analysis pipeline than typical latent model approaches to understanding structure.

Although network analysis has been previously applied to psychosis in analyses that include paranoia as one of the symptoms [10] (reviews in Isvoranu et al. [11, 12]), to our knowledge, network analysis has only been specifically applied to understanding paranoia on one previous occasion: a single patient time series study looking at the network change in symptoms over time [13].

Consequently, we used network analysis to re-examine the same epidemiological dataset used by Bebbington et al. [3] to investigate the symptom-level network structure of paranoia in the general population. We also aimed to test how closely network sub-clusters, identified by network community analysis, would match the factor structure derived from the original analysis using latent variable models, to test whether a similar structure would arise using a markedly different method of analysis. If the original model can be found using exploratory unsupervised techniques, then the evidence supporting the model becomes more robust.

## Methods

### Sample

Following Bebbington et al. [3], data were taken from the British National Psychiatric Morbidity Survey conducted in the year 2000. The Psychiatric Morbidity Survey is conducted approximately every 7 years in the UK and includes a representative population sample drawn from across the country. Details of the sampling are reported in full in Singleton et al. [4]. The original researchers generated a list of 15,804 households and interviewers visited each address and those which were private households with at least one person age 16–74 years were invited to participate. Interviews were conducted in two phases. An initial interview that contained the majority of mental health measures conducted by trained interviewers from the Office for National Statistics. People who screened positive for psychosis and/or personality disorder were invited for a second interview by clinicians to establish a diagnosis. The final dataset contains data for 8576 individuals.

Although no specific assessment for paranoia was included, Bebbington et al. [3] chose the year 2000 Psychiatric Morbidity Survey dataset as it included both the Psychosis Screening Questionnaire (PSQ) and the questionnaire version of the Structured Clinical Interview for DSM-IV Axis II Disorders (SCID-II [14]). The SCID-II contained items from personality disorder assessments which, with the PSQ, covered a comprehensive range of paranoid experiences. Specifically, their analysis included items 2, 3, 3a and 3b from the Psychosis Screening Questionnaire (PSQ [15]) and items 2, 3, 4, 6, 10, 25, 26, 27, 28, 33 and 35 from the SCID-II. These exact same items were included in the analysis for the present study. PSQ items were scaled from 0 to 2 (0 = absent, 1 = unsure, 2 = present) and SCID-II items were scaled from 0 (absent) to 1 (present). “Don’t know” or “Don’t understand” responses were coded as missing. SCID-II items had an average of 2.95% missing data, PSQ items an average of 0.06% missing data. Overall item endorsement ranged from 6.72 to 28.67% (mean = 18.38%; SD = 7.02%). No special considerations for missing data were mentioned in the original Bebbington et al. [3] study and so missing data were not removed. Full item endorsement statistics are reported in Table S1 of the supplementary information.

### Network analysis

We constructed a network where each of the items was represented in the network as nodes and the correlation between items as edges (the ‘links’ in the network). The network was constructed using the *qgraph* package [16] (version 1.4.4) for the statistical programming language *R* (version 3.4.2) defaulting to pairwise estimation for missing data. Analysis was conducted on a 64-bit x86 linux platform. The dataset is available on application from the UK Data Service [17]. The *R* script to conduct the analyses reported in this study is available online at http://osf.io/b9ngx.

A polychloric correlation matrix (that computes correlations for ordinal variables) for all selected items was calculated and this formed the basis of the network structure. The final network structure was derived using a Graphical Least Absolute Shrinkage and Selection Operator (glasso) method [18] that estimated a penalized maximum likelihood solution based on the Extended Bayesian Information Criterion (EBIC). This produces an optimal network structure that is highly likely to reflect the genuine structure in the population [19–21]. The EBIC glasso network estimation requires a tuning parameter (gamma) which was set to 0.5 which is recommended by Foygel and Drton [19] as a conservative threshold for keeping edges that likely represent genuine relationships found in the population. In the network visualisations, the strengths of associations are represented by the thickness of the lines.

Networks were displayed using the Fruchterman-Reingold algorithm [22] where nodes with the strongest connections and preferentially placed at the centre of the network and those with weaker connections towards the outside.

### Centrality metrics

For each network, we also computed centrality metrics for every node. These included betweenness centrality (the number of times a particular node lies on the shortest path between two other nodes), closeness centrality (the mean distance from a node to all other network nodes) and strength centrality (the sum of the strength of edges connected to the node), each reflecting the relative ‘importance’ of a particular node in terms of its centrality in mediating associations between nodes and maintaining the structure of the network. To assess the stability of centrality metrics we used an *m out of n* bootstrap method where increasing numbers of cases are removed from the dataset and centrality metrics recalculated giving a correlation stability coefficient using the *bootnet* package [23] (version 1.0.1) for *R* specifying 2500 permutations.

### Network sub-community identification

Network communities or clusters were identified using two approaches. The first was the spinglass algorithm implemented in the *igraph* package for *R* [24] (version 1.1.2) that uses a spinglass model and simulated annealing to identify sets of nodes with many edges inside the community and few edges outside it. The second was the *EGA* package for *R* [25, 26] (version 0.2) that uses a random walk algorithm to identify sub-communities in networks which has been shown to show greater accuracy in identifying dimensions in psychometric data than both exploratory [25] and confirmatory [27] factor analytic methods.

## Results

Initial attempts to generate a polychloric correlation network with the entire sample resulted in a non-positive definite matrix error. This was caused by variables representing items 3a and 3b on the Psychosis Screening Questionnaire which are only asked if item 3 is answered positively, meaning these variables are not independent. Because items 3a (“Have there been times when you felt that people were deliberately acting to harm you or your interests”) and 3b (“Have there been times when you felt that a group of people was plotting to cause you serious harm or injury”) are elaborations of item 3 (“Over the past year, have there been times when you felt that people were against you?”) items 3a and 3b were eliminated from the analysis and therefore the final network analysis was conducted on items 2 and 3 from the Psychosis Screening Questionnaire and items 2, 3, 4, 6, 10, 25, 26, 27, 28, 33 and 35 from the SCID-II.

### Network structure

**Fig. 1:**
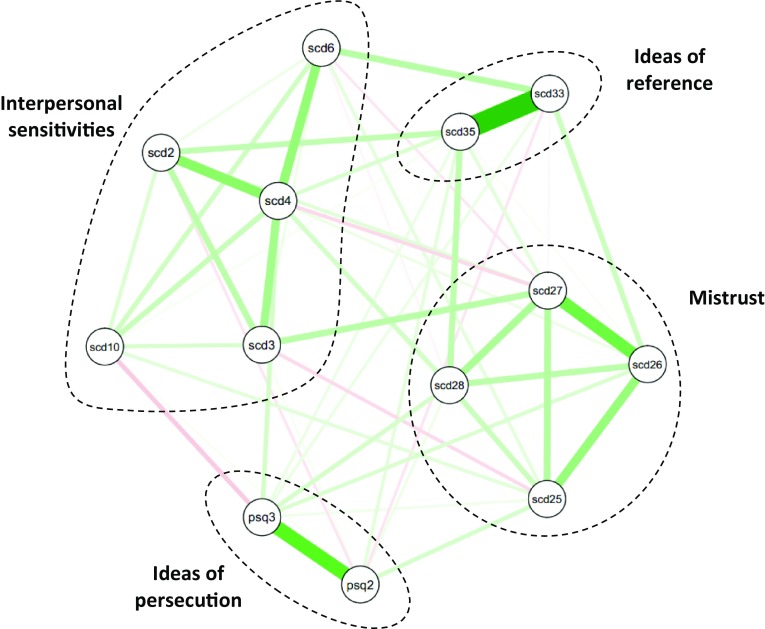
Glasso network structure of paranoia items from the British National Psychiatric Morbidity Survey (2000) with sub-communities identified by spinglass and EGA analyses highlighted. Item key shown in Table 1

The glasso network is displayed in Fig. 1 and the figure key for scale items is shown in Table 1. Results from the bootstrapped centrality stability analysis showed generally robust centrality estimates as cases are removed from the network. Bootstrapped difference tests for edge-weights and centrality demonstrated that a high proportion of comparisons were statistically significant. Full details are reported in the supplementary material. Correlation stability coefficients computed for the centrality metrics (betweenness = 0.206, closeness = 0.283, strength = 0.672)^1^, showed that closeness and strength centrality metrics surpassed the recommended value of 0.25 [23] although the betweenness centrality metric did not, suggesting it should be interpreted with caution.

**Table 1:** Items from British National Psychiatric Morbidity Survey (2000) included in the network analysis grouped by spinglass and EGA analysis

Label	Scale item
*Community 1* (*Interpersonal sensitivities*)	
scd2	Do you avoid getting involved with people unless you are certain they will like you?
scd3	Do you find it hard to be ‘open’ even with people you are close to?
scd4	Do you often worry about being criticised or rejected in social situations?
scd6	Do you believe that you’re not as good, as smart, or as attractive as most other people?
scd10	Do you find it hard to disagree with people even when you think they are wrong?
*Community 2* (*Mistrust*)	
scd25	Do you often have to keep an eye out to stop people from using you or hurting you?
scd26	Do you spend a lot of time wondering if you can trust your friends or the people you work with?
scd27	Do you find that it is best not to let other people know much about you because they will use it against you?
scd28	Do you often detect hidden threats or insults in things people say or do?
*Community 3* (*Ideas of reference*)	
scd33	When in public and see people talking, do you often feel that they are talking about you?
scd35	When you are around people, do you often get the feeling that you are being watched or stared at?
*Community 4* (*Ideas of persecution*)	
psq2	Have you felt that your thoughts were directly interfered with or controlled by some outside force or person?
psq3	Over the past year, have there been times when you felt that people were against you?

### Centrality metrics

**Fig. 2:**
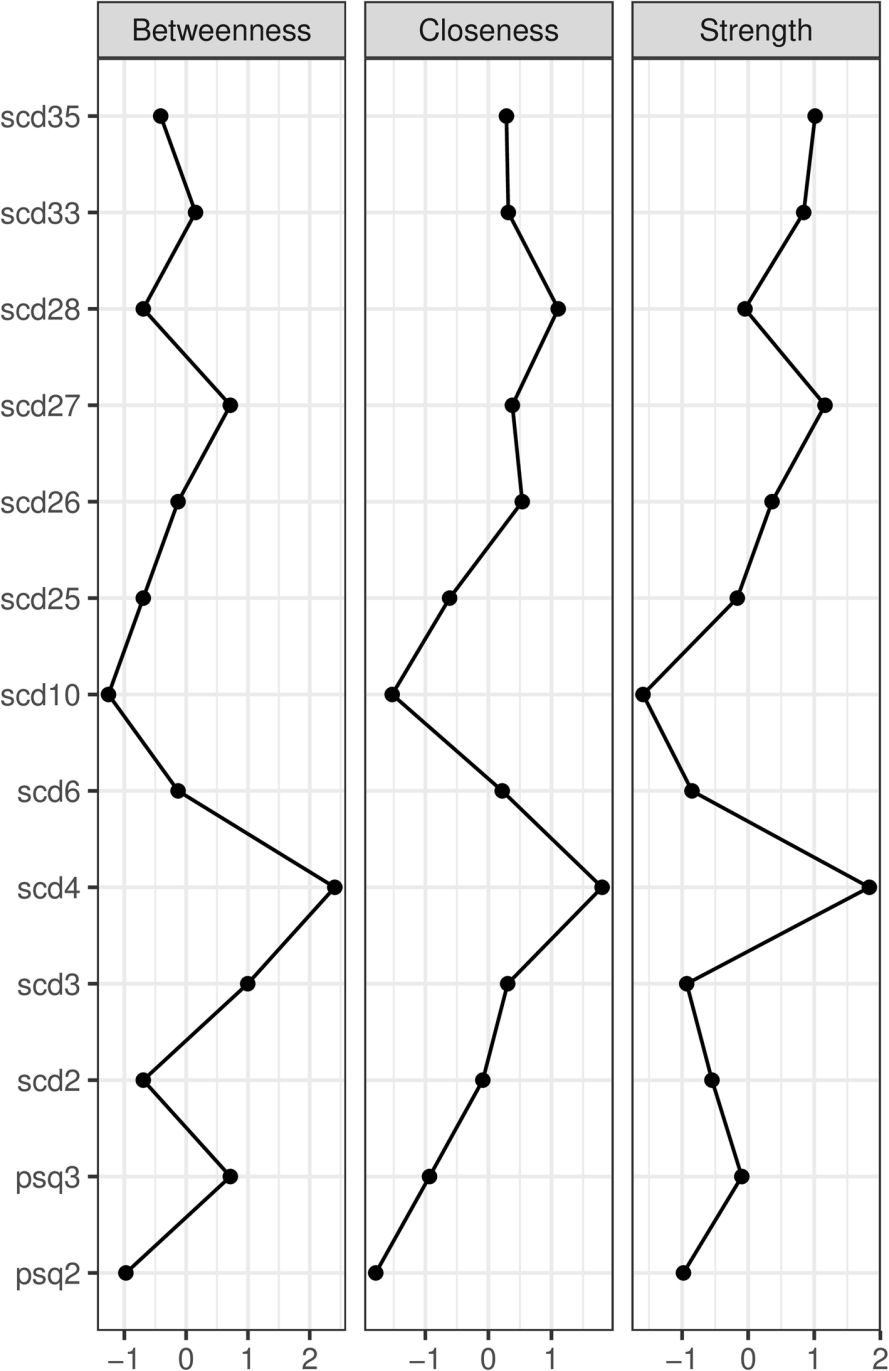
Betweenness, closeness and strength centrality metrics for the network nodes

The graph displaying centrality metrics is shown in Fig. 2. The network is highly interconnected but with some more central nodes present in the network. The most central node across all three measures of betweenness, closeness and strength was scd4 (“Do you often worry about being criticised or rejected in social situations?”) highlighting the centrality of worry in maintaining paranoia, followed by item scd27 (“Do you find that it is best not to let other people know much about you because they will use it against you?”) indicating mistrust of others’ intentions. Among the least central nodes were scd10 (“Do you find it hard to disagree with people even when you think they are wrong?”) and psq2 (“Have you felt that your thoughts were directly interfered with or controlled by some outside force or person?”).

### Network sub-community identification

The spinglass and EGA community analyses identified identical sub-communities or clusters in the overall network and so are presented here together. Figure 1 shows the glasso network map with the sub-communities highlighted and Table 1 shows the individual items by sub-community grouping. Notably, the sub-communities exactly matched the apriori paranoia theme groupings from Bebbington et al. [3] (minus the items eliminated in our analysis for reasons of non-independence) and closely overlapped with the four factor first order model derived from their latent class analysis (the only differences from their model were that item scd3 was grouped into ‘interpersonal sensitivities’ instead of ‘mistrust’ and item scd28 was grouped into ‘mistrust’ instead of ‘ideas of reference’). Our analysis indicated that the same four themes constituted the structure of paranoia when identified by network community analysis: interpersonal sensitivities, mistrust, ideas of reference and ideas of persecution. We also calculated the mean centrality metrics for each sub-community based on individual item centrality metrics which are displayed in Table 2. The ‘Mistrust’ and ‘Ideas of reference’ were the most central to the network as a whole.

**Table 2:** Mean centrality metrics per network sub-community

	Betweenness	Closeness	Strength	Overall mean
Interpersonal sensitivities	0.26	0.14	− 0.42	− 0.004
Mistrust	− 0.20	0.35	0.33	0.160
Ideas of reference	− 0.13	0.30	0.93	0.366
Ideas of persecution	− 0.13	− 1.36	− 0.54	− 0.676

## Discussion

In this study we report a network analysis of paranoia in the general population using data from the year 2000 British National Psychiatric Morbidity Survey. Sub-community analysis identified four sub-communities in the wider network of paranoia that matched the four factors identified by Bebbington et al. [3], namely interpersonal sensitivities, mistrust, ideas of reference and ideas of persecution.

In terms of the potential importance of individual nodes, the most central was the item “Do you often worry about being criticised or rejected in social situations?” reflecting the well-established role of worry in maintaining paranoia [28]. Interestingly, the subsequent most central item was “Do you find that it is best not to let other people know much about you because they will use it against you?” suggesting a negative social representation of others [29]. One of the advantages of network analysis is that it suggests potential targets for intervention, given that it is possible to identify symptoms that are plausible candidates for change with potential for dismantling the wider network. Although worry has been an effective target for intervention [28], it is notable that interventions for paranoia to date have rarely considered social representations (‘schemas’) of others as a relevant focus. This is despite the fact that ‘negative-other’ schemas are some of the strongest predictors of paranoia [30–32] and there is now initial experimental evidence that suggests a negative representation of others is an important factor alongside an exaggerated sense of personal danger in paranoia [33].

Using two separate methods to identify network sub-communities (network sub-clusters) our analysis identified the same four themes as the Bebbington et al. [3] analysis, providing further evidence for these four factors comprising the ‘structure’ of paranoia in terms of potential components. Freeman et al. [2] and Bebbington et al. [3] have suggested that paranoia shows a hierarchical structure, in that the rarest and least endorsed ideas are associated with a greater number of total endorsed paranoid ideas. From this perspective, clinical paranoia is built on a base of more everyday concerns about safety and social evaluation, with delusional paranoia topping the hierarchy.

Alternatively, a network approach to psychopathology envisions symptoms as forming into self-sustaining networks, with individual symptom nodes differing in their ability to activate and influence other symptoms [7]. More central symptoms are considered more likely to activate other symptoms as they themselves become more active, whereas less central symptoms will have little effect on other symptoms as they become active. In this approach, paranoia is best thought of not as a hierarchy, but as a heterarchy, where different structures (including hierarchies) may exist within the same network.

We suggest the latter is more likely in this case. There may be a common hierarchical structure in the population in terms of frequency of endorsement, but that may not fully describe the way activation spreads through the network or the importance of certain sub-communities for maintaining steady states of symptom activation. Indeed, out of the four sub-communities identified in the analysis, ‘mistrust’ and ‘ideas of reference’ had the highest average centrality metrics, suggesting that they may be the most important in terms of maintaining a stable self-sustaining paranoid state. The two other sub-communities ‘interpersonal sensitivities’ and ‘ideas of persecution’—represent the experiences least and most reflective of delusional paranoia, but may be least important in terms of maintaining steady state paranoia. Indeed, this is consistent with evidence from experience sampling studies that shows that the intensity of frank paranoid delusions fluctuates quite considerably even on a day-to-day basis [34, 35]. One potential hypothesis from these findings would be that ‘mistrust’ and ‘ideas of reference’ symptoms are less likely to fluctuate when activated than ‘interpersonal sensitivities’ and ‘ideas of persecution’ sub-communities. However, it is also worth noting that the most central node (related to worry) formed part of one of the least central sub-communities (‘interpersonal sensitivities’) again suggesting that a simple hierarchical structure is not sufficient to understand the structure of paranoia.

It is also worth noting the methodological novelty of this study. Namely, using network analysis to re-examine data from which components were previously identified using latent variable methods. Although the sub-communities we identified in this study very closely matched the latent variables identified in Bebbington et al. [3], it is conceivable that in some datasets network analyses might generate a network which does not match the outcome of factor analysis or similar methods. This issue of interpretation in these differing scenarios has yet to be resolved methodologically [36], although we suggest it is likely that if a network analysis replicates a previously identified structure by other methods this provides good confirmatory evidence for a general underlying structure. If it does not, the extent to which the network analysis is showing an alternative structure that indicates interactions—its dynamic structure perhaps—rather than common causes, may need to tested through experimental analysis of the components and their varying relationships.

In terms of limitations, this study is subject to similar limitations identified in Bebbington et al. [3], namely the use of self-report responses, the extraction of paranoia-relevant items from non-paranoia specific measures and differences in question framing between the Psychosis Screening Questionnaire (which asks about experiences within the past year) and the SCID-II (which asks about a general tendency to think in certain ways).

However, there are also limitations specific to the network analysis in this study. One aspect is that this study included two less items from the Psychosis Screening Questionnaire than the original analysis because they depended on an earlier item and were therefore non-independent. It is not clear how the authors of the original study dealt with this, considering that similar considerations apply to the analysis methods they used, but this does mean the data was possibly not completely identical between studies.

Network analysis produces candidates for causal interactions between symptoms. However, strong inferences about cause cannot be made purely on the basis of these findings. Indeed, even with genuine causal relationships, interactions may evolve over different timescales which are not well represented in the network structure from the cross-sectional data presented here [7] and time series data are needed to fully address this. The same consideration holds for interpretation of more central nodes as the most important or influential in terms of sustaining network activation, or in terms of identifying effective points of clinical intervention. Although these hypotheses are plausible [8], conclusions about causality and effective points of intervention still ultimately rest on experimental intervention studies.

In conclusion, this study reports additional evidence to support four key components underlying the structure of paranoia in the general population uncovered using an alternative to latent variable analysis. This study additionally highlights symptoms, either individually or as communities, that are central to maintaining the symptom network and may be plausible candidates for intervention.

## Electronic supplementary material

Below is the link to the electronic supplementary material.

Supplementary material 1 (DOCX 3822 KB)

## Footnotes

1 As a bootstrap analysis, the stability coefficients rely on an element of random sampling. On some runs, all centrality metrics are above cut-off. However, we report the most conservative metrics obtained here which should reflect the minimum metric stability.
