# Supplementary material for: The network structure of paranoia in the general population
Source: Soc Psychiatry Psychiatr Epidemiol. 2018 Feb 9;53(7):737–44. doi: 10.1007/s00127-018-1487-0 (PMC6003969; doi:10.1007/s00127-018-1487-0)
Supplement: Supplementary file 1 — Supplementary material 1 (DOCX 3822 KB) [file 127_2018_1487_MOESM1_ESM.docx]

**Supplementary Materials**

|  | *pd2* | *pd3* | *pd4* | *pd6* | *pd10* | *pd25* |
| --- | --- | --- | --- | --- | --- | --- |
| Yes | 1102  (12.8%) | 1788  (20.8%) | 2329 (27.1%) | 2179 (25.4%) | 1754  (20.4%) | 2460  (28.7%) |
| No | 7201 (83.9%) | 6563 (76.5%) | 6017 (70.1%) | 6111 (71.2%) | 6589 (76.8%) | 5868 (68.4%) |
| Missing | 277  (3.2%) | 229  (2.7%) | 234 (2.7%) | 290 (3.4%) | 237  (2.8%) | 252 (2.9%) |

|  | *pd26* | *pd27* | *pd28* | *pd33* | *pd35* |
| --- | --- | --- | --- | --- | --- |
| Yes | 1352 (15.8%) | 1918 (22.4%) | 1579 (18.4%) | 577 (6.7%) | 853 (9.9%) |
| No | 6960 (81.1%) | 6370 (74.2%) | 6734 (78.5%) | 7778 (90.7%) | 7515 (87.6%) |
| Missing | 268 (3.1%) | 292 (3.4%) | 267 (3.1%) | 225 (2.6%) | 212 (2.5%) |

|  | *psq2* | *psq3* |
| --- | --- | --- |
| Yes | 805 (9.4%) | 1810 (21.1%) |
| Unsure | 64 (0.7%) | 76 (0.9%) |
| No | 7704 (89.8%) | 6691 (78.0%) |
| Missing | 7 (0.08%) | 3 (0.03%) |

**Table S1 Item Endorsement Frequencies**

**Stability Analyses**

We report stability tests which refer to the extent to which the network and its parameters, and hence conclusions drawn from them, remain robust after systematic variation and re-sampling using bootstrap methods – as described by and implemented in the *bootnet* package for R (Epskamp et al., 2016). All bootstrap analyses are run using 2500 iterations.

*Confidence intervals for edge-weights*

The edges (connections) between nodes have a weight. Using bootstrap methods, a 95% CI around the edge weights can be constructed. The graph of the bootstrap analysis is displayed in Figure S1. The red line indicates edge weight and the grey borders indicate the extent of the bootstrapped confidence intervals. Wide confidence intervals indicate low stability and confidence intervals that remain close to the value indicate high stability. Overlapping confidence intervals signify that edge weights are unlikely to significantly differ from one-another.


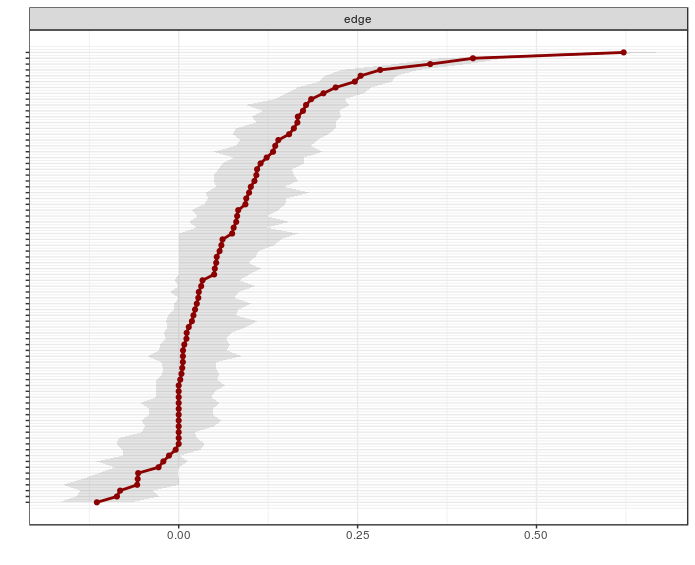


**Figure S1. Accuracy of edge weights**

Accuracy of edges estimated with bootstrapped 95% confidence intervals. The smaller confidence intervals indicate more accurate edge estimates.

*Significant differences in node strength*

Bootstrap confidence interval tests can also estimate significant differences between the strength of any node pairing. These results are displayed in Figure S2 showing a large proportion of the node strength comparisons are significantly different (p < 0.05).


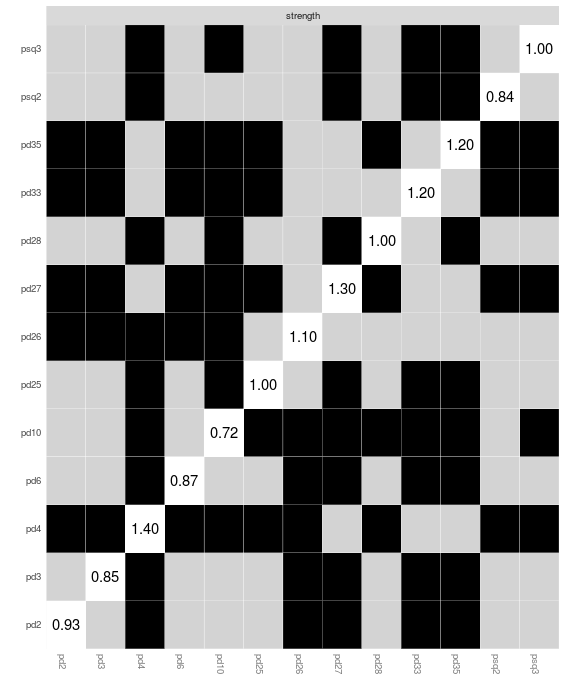


**Figure S2. Bootstrapped difference tests for node strength**

Black represents a significant difference in node strength for each pairing, grey a non-significant difference, white the node strength value.

*Significant differences in edge-weights*

Bootstrapped difference tests can also be applied to edge-weight comparisons. Figure S3 displays significant differences between all edge-weight pairings, showing a high proportion of significant differences.


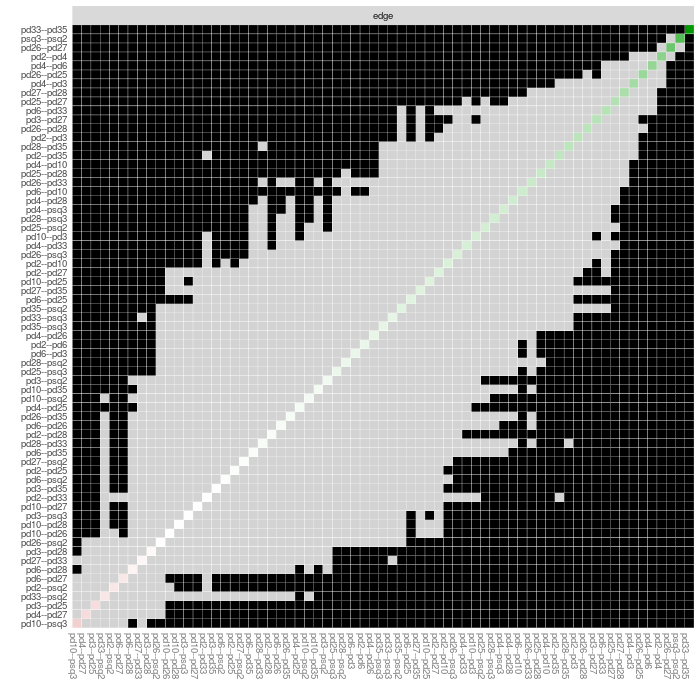


**Figure S3. Bootstrapped edge weights difference test**

Black represents a significant difference between edge weight pairings, grey a non-significant difference.

*Stability of centrality metrics*

The stability of centrality metrics can be tested by correlating the metrics obtained from the full sample with metrics obtained after systematically removing increasing numbers of cases from the analysis. Graph Figure S3 shows the stability of the betweenness, closeness, and strength metrics during this process. Please note: As a bootstrap analysis, the stability coefficients rely on an element of random sampling. On some runs, centrality metrics show markedly better performance. However, we report the most conservative metrics obtained here which should reflect the minimum metric stability.

**
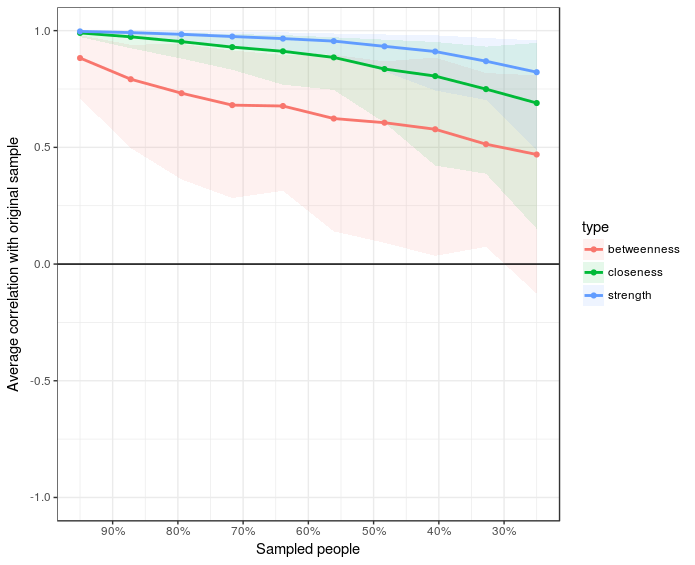
**

**Figure S4. Centrality Metrics Stability**

Correlation of the original centrality metrics with metrics calculated with increasing numbers of randomly removed participants.
